# Development of Computerized Adaptive Testing for Emotion Regulation

**DOI:** 10.3389/fpsyg.2020.561358

**Published:** 2020-12-01

**Authors:** Lingling Xu, Ruyi Jin, Feifei Huang, Yanhui Zhou, Zonglong Li, Minqiang Zhang

**Affiliations:** ^1^School of Psychology, South China Normal University, Guangzhou, China; ^2^Key Laboratory of Brain, Cognition and Education Sciences (South China Normal University), Ministry of Education, Guangzhou, China; ^3^Center for Studies of Psychological Application, South China Normal University, Guangzhou, China; ^4^Guangdong Key Laboratory of Mental Health and Cognitive Science, South China Normal University, Guangzhou, China

**Keywords:** emotion regulation, computerized adaptive testing, item response theory, item bank, measurement

## Abstract

Emotion regulation (ER) plays a vital role in individuals’ well-being and successful functioning. In this study, we attempted to develop a computerized adaptive testing (CAT) to efficiently evaluate ER, namely the CAT-ER. The initial CAT-ER item bank comprised 154 items from six commonly used ER scales, which were completed by 887 participants recruited in China. We conducted unidimensionality testing, item response theory (IRT) model comparison and selection, and IRT item analysis including local independence, item fit, differential item functioning, and item discrimination. Sixty-three items with good psychometric properties were retained in the final CAT-ER. Then, two CAT simulation studies were implemented to assess the CAT-ER, which revealed that the CAT-ER developed in this study performed reasonably well, considering that it greatly lessened the test items and time without losing measurement accuracy.

## Introduction

Emotion regulation (ER) has received substantial and increased attention in psychology and related fields (Campos et al., 2011), as its processes affect an individual’s experience of positive and negative emotions, interpersonal relationships, as well as physical health (Gross and John, 2003). ER has long been regarded as a crucial mechanism underlying physical and mental health. Research into ER skills is linked to psychological well-being, social relations, and emotional functioning (Gross and John, 2003; Balzarotti et al., 2010; Gullone and Taffe, 2011). Both theoretical and empirical findings indicate ER’s critical role in many areas of psychopathology research, involving anxiety (Mennin et al., 2009), eating (Lavender et al., 2014), and personality disorders (Bornovalova et al., 2008). Therefore, it is important to make an accurate evaluation and diagnosis of those with ER difficulties and offer timely treatment.

Recently, there has been a proliferation of self-report ER measures (Aldao et al., 2010; Megreya et al., 2018), such as the Difficulties in Emotion Regulation Scale (DERS; Gratz and Roemer, 2004), Negative Mood Regulation Scale (NMR; Catanzaro and Mearns, 1990), Regulatory Emotional Self-Efficacy Scale (RESE; Caprara et al., 2008), Emotion Regulation Questionnaire (ERQ; Gross and John, 2003), Trait Meta-Mood Scale (TMMS; Salovey et al., 1995), and Cognitive Emotion Regulation Questionnaire (CERQ; Garnefski et al., 2001). Previous studies (Garnefski et al., 2001; Gross and John, 2003; Gratz and Roemer, 2004; Caprara et al., 2008) suggest that each measure mentioned above is formed based on the same underlying structure (i.e., ER). Example items of these measure can be seen in the “Measures” section. The description based on the content of the individual items may aid in better reflecting the same structure of these measures to a certain extent. Additionally, critical evidence from several meta-analytic reviews indicates each scale measures and evaluates the same underlying ER structure. For example, Naragon-Gainey et al. (2017) showed that self-report ER measures, including the ERQ, DERS, and CERQ, have been used to examine the underlying structure of common ER and evaluate it in light of the theoretical models of ER. Aldao et al.’s (2010) study also found that the aforementioned self-report scales were used to measure and evaluate ER. Furthermore, several recent studies reported that the DERS, ERQ, RESE, CERQ, NMR, and TMMS have been incorporated into a common structural model of ER (Gross, 2014; Megreya et al., 2018).

To date, these self-report ER measures have only been applied using the paper-and-pencil (P&P) method. P&P questionnaires are usually onerous for the subjects, because all items need to be administered regardless of whether they are related or not. For instance, certain items may be highly difficult or extremely easy for the individual, relying on their level of the construct being measured (e.g., ER). Meanwhile, the collection of subjects’ responses employing P&P measures demands subsequent data entry and scoring calculations, both of which warrant considerable resources and expertise.

One way to address the abovementioned problems is by performing adaptive testing. Computerized adaptive testing (CAT) typically involves an item-level adaptive test, a form of testing that employs item response theory (IRT) to build an item bank, and automatically chooses appropriate items from it according to the subject’s ability level, which is updated based on the subject’s responses to each item. This process continues until the test taker’s theta reaches satisfactory accuracy. In contrast to P&P testing, the primary advantage of CAT is that it demands fewer test items and takes less time to achieve similarly accurate scores, lessening administration burden and ensuring subjects’ motivation. Additionally, the test developer can preset the degree of measurement accuracy demanded in a CAT design. More importantly, CAT automates the administration of data, scoring, and reporting, which enables the results to be integrated into feedback and treatment immediately.

Given these benefits, CAT and item banking are becoming increasingly popular in the field of ability measurement and psychology, including some large-scale tests such as the Graduate Record Examination (GRE) and Test of English as a Foreign Language (TOEFL), as well as tests to assess anxiety (Walter et al., 2007) and depression (Gardner et al., 2004). Considering the drawbacks of P&P testing and the advantages of CAT, this study attempted to offer a novel ER evaluation technique by employing CAT as the measurement method with a Chinese sample. More specifically, this study addressed some existing critical issues. First, a calibrated item bank with good psychometric properties was developed in this study. Second, the reliability and validity of the CAT-ER were assessed using two simulation studies in different stopping rules. Third, we provide recommendations for applicators who plan to put adaptive testing into use.

## Materials and Methods

### Participants

A total of 887 Chinese adolescents and adults living in 29 randomly selected cities in China, aged between 16 and 64 years (median = 30.612 years, *SD* = 13.527), participated in this study (83.3% response rate). Respondents were recruited from July to September 2019. Table 1 contains detailed demographic characteristics. The sample included 549 females (61.9%) and 338 males (38.1%). There were 448 (50.5%) participants under 25 years old and 439 (49.5%) aged 25 and above. The participants came from urban (55.1%) and rural (44.9%) regions. Volunteers anonymously completed the demographic questions and self-report measures online. The purpose of this study, assurances of confidentiality, and participants’ rights were explained to them. All subjects provided written informed consent and were paid for their participation. The current study was approved by the local Ethics Committee of the School of Psychology, South China Normal University.

**TABLE 1:** Demographic characteristics (*N* = 887).

Variables	Category	Frequency	Percent (%)
Gender	Male	338	38.1
	Female	549	61.9
Age	Under 25 years	448	50.5
	25 and above	439	49.5
Region	Rural	398	44.9
	Urban	489	55.1

### Measures

Drawing on the findings from prior studies, the 154 items from six frequently employed Chinese-versions of self-report ER measures were used to build the initial CAT-ER item bank (Table 2). The ER measures used in this study included the DERS (Li and Wu, 2018), TMMS (Li et al., 2002), RESE (Wen et al., 2009), ERQ (Li and Wu, 2018), CERQ (Zhu et al., 2008), and NMR (Wang et al., 2017). Example items of the DERS are “I am clear about my feelings” and “When I’m upset, I believe my emotions are valid and important.” Example items of the TMMS are “I try to think of good thought no matter how badly I feel” and “No matter how badly I feel, I try to think about pleasant things.” Example items of the RESE are “Express enjoyment freely at parties” and “Avoid flying off the handle when you get angry.” Example items of the ERQ are “I keep my emotions to myself” and “I control my emotions by not expressing them.” Example items of the CERQ are “I think that I have to accept the situation” and “I think about how to change the situation.” Example items of the NMR are “I can do something to feel better” and “I can find a way to relax.” Each of the five measures (i.e., DERS, TMMS, RESE, CERQ, NMR) is scored on a 5-point Likert scale, and the ERQ is scored on a 7-point Likert scale. Higher scores for several items of the DERS, NMR, and RESE indicate greater difficulties in ER. In this study, we attempted to measure the ability of individual successful ER, reverse-coded items thus needed to be forward coded. Through the above approach, a higher total score of these measures denoted a more successful ER performance.

**TABLE 2:** Items from six scales.

Scale	Number of items	Items
DERS	36	DERS-1, 2, 3, 4, 5, 6, 7, 8, 9, 10, 11, 12, 13, 14, 15, 16, 17, 18, 19, 20, 21, 22, 23, 24, 25, 26, 27, 28, 29, 30, 31, 32, 33, 34, 35, and DERS-36
TMMS	30	TMMS-1, 2, 3, 4, 5, 6, 7, 8, 9, 10, 11, 12, 13, 14, 15, 16, 17, 18, 19, 20, 21, 22, 23, 24, 25, 26, 27, 28, 29, and TMMS-30
RESE	12	RESE-1, 2, 3, 4, 5, 6, 7, 8, 9, 10, 11, and RESE-12
ERQ	10	ERQ-1, 2, 3, 4, 5, 6, 7, 8, 9, and ERQ-10
CERQ	36	CERQ-1, 2, 3, 4, 5, 6, 7, 8, 9, 10, 11, 12, 13, 14, 15, 16, 17, 18, 19, 20, 21, 22, 23, 24, 25, 26, 27, 28, 29, 30, 31, 32, 33, 34, 35, and CERQ-36
NMR	30	NMR-1, 2, 3, 4, 5, 6, 7, 8, 9, 10, 11, 12, 13, 14, 15, 16, 17, 18, 19, 20, 21, 22, 23, 24, 25, 26, 27, 28, 29, and NMR-30

*DERS, Difficulties in Emotion Regulation Scale; TMMS, Trait Meta-Mood Scale; RESE, Regulatory Emotional Self-Efficacy Scale; ERQ, Emotion Regulation Questionnaire; CERQ, Cognitive Emotion Regulation Questionnaire; NMR, Negative Mood Regulation Scale.*

We implemented two types of analyses to develop the CAT-ER based on the IRT. The first analysis was to satisfy the psychometric requirements of the CAT-ER, and the second was to simulate the CAT adaptively using existing item responses and simulated responses. Friedman et al. (2010) stated that overfitting and overly optimistic results might appear when the same sample is used to estimate item parameters and simulate CAT studies. Therefore, the real 887 subjects’ responses collected in advance were randomly divided into a training and a test set. Reise and Yu (1990) suggested, a sample size of at least 500 is considered enough for acceptable item parameter estimates for unidimensional polytomous IRT models through simulation studies in the item bank calibration. Therefore, we applied the real 500 subjects’ responses in constructing the CAT-ER item bank and used the real 387 subjects’ responses to simulate the CAT-ER to confirm the precision and validity of the algorithm. Furthermore, to test the performance of this method on the test set after iterating all item reduction methods on the training set, the real 387 subjects’ responses also were used to validate the psychometric properties of the final item set of the CAT-ER.

### Construction of CAT-ER Item Bank

#### Unidimensionality

In IRT, unidimensionality is an important assumption, and an item bank is deemed unidimensional when a subject’s item responses derives from the subject’s actual trait level that the item measures and not from other elements. Therefore, there is a need to assess the unidimensionality of the item bank for IRT applications (Reise et al., 2007). A factor analytical framework and an IRT framework were used to evaluate unidimensionality.

First, data were randomly divided into two sets (*n* = 250 each), one for exploratory factor analysis (EFA) and the other for confirmatory factor analysis (CFA). In EFA, we used parallel analysis as the criterion to estimate the number of meaningful factors. Items with factor loadings over 0.30 that were significant at *p* = 0.05 were considered for retention in the development of the item bank. In CFA, the root mean square error of approximation (RMSEA), comparative fit index (CFI), and Tucker-Lewis index (TLI) were utilized to evaluate the goodness of fit. Researchers have pointed out that RMSEA ≤ 0.08, CFI ≥ 0.90, and TLI ≥ 0.90 denote an acceptable fit (Kline, 2010). Next, we calculated the proportion of total variance attributable to a general factor with omega hierarchical (*ω*_*h*_).*ω*_*h*_≥0.70, indicating that the item set is fully unidimensional (Reise et al., 2013). Moreover, the proportion of explained common variance (ECV) is also a useful index for determining the importance of the general factor. The ECV cut-off value in a bifactor model is 60%, and a higher value means better performance (Reise et al., 2013).

Additionally, unidimensionality was examined by a descriptive analysis of the standardized residuals of item responses for the IRT model. A goodness-of-fit test was conducted from a fitted IRT model to detect violations of the unidimensionality hypothesis in the test data (Finch and Habing, 2007). The *M*_2_ statistic was employed to test the overall goodness of fit of a parametric model (Maydeu-Olivares and Joe, 2006).

#### IRT Model Selection

In the IRT framework, a suitable model is needed for parameter estimation. Some frequently employed multi-index IRT models for polytomous data involve the graded response model (GRM; Samejima, 1969), the generalized partial credit model (GPCM; Muraki, 1992), and the nominal response model (NRM; Bock, 1972). We chose an optimal model for further analysis based on the following test-level model fitting indexes: −2 log-likelihood (−2LL; Spiegelhalter et al., 1998), Akaike’s information criteria (AIC; Akaike, 1974), Bayesian information criterion (BIC; Schwarz, 1978), and *M*_2_ statistic (Maydeu-Olivares and Joe, 2006). Smaller values of these indexes reflect a better model fit. The goodness of fit (*M*_2_ statistic) was not statistically significant, indicating that the data fit well in the model.

#### Local Independence

To ensure that parameter estimates are not excessively distorted due to related item pairs, we evaluated local dependence in the IRT framework with residual correlations and Yen’s (1993) Q3 statistic. Based on a prior study (Flens et al., 2017), residual correlation values of 0.20 or greater and Yen’s Q3 statistic values of 0.36 or higher were deemed large and unexpected.

#### Item Fit

Item fit was investigated using the *S*-χ^2^ statistic (Kang and Chen, 2008). Items with a *S*-χ^2^ *p* < 0.01 were considered to have a poor fit and were thus removed from the item bank.

#### Differential Item Functioning

Ordinal logistic regression (Crane et al., 2006) was applied to assess Differential Item Functioning (DIF) using the R package “lordif” (Choi, 2015). Change in McFadden’s pseudo-*R*^2^ was employed to assess the effect size, and the hypothesis of no DIF was rejected when Δ*R*^2^ > 0.02 and *p* > 0.05 (Flens et al., 2017), and such items were excluded. We assessed DIF for gender, age, and region groups.

#### Item Discrimination

Researchers suggest that a discrimination value of 0.50–2.50 indicates an acceptable IRT discrimination (Chang and Ying, 1996). In this study, items with poor discrimination values (i.e., discrimination value < 0.50) were removed from the item bank.

Based on the steps detailed above, all items that satisfied the psychometric requirements were retained to develop the final item set of the CAT-ER. To test the performance of this method on the test set after iterating all item reduction methods on the training set, a similar evaluation (i.e., unidimensionality, local independence, item fit and DIF) was conducted on the test set. We used the R package “psych” for EFA and bifactor analysis, and Mplus 7.0 (Muthén and Muthén, 2012) for CFA. The IRT analyses (involving unidimensionality, model selection, local independence, item fit, and item discrimination) were performed using the R package “mirt” (Chalmers, 2012).

### Simulation of the CAT-ER

The CAT simulation was implemented after the final item set of the CAT-ER was built. Based on the real-item parameters of the CAT-ER, the performance of the CAT-ER using simulated data in various ER levels was simulated to assess its rationality. The abilities of 1,000 virtual persons were generated in random from *N* (0,1); this sample was considered to represent the true theta values. The advantage of simulating new thetas includes the ability to reach full ranges of the subjects with various theta values.

There were four stages in the CAT simulation, including the initial, test, stop, and final stages (Magis and Raiche, 2012). In the initial stage, the first item was chosen randomly from the final item set of the CAT-ER; then, the subject’s response was simulated based on the true theta value simulated before and the randomly chosen initial item. Furthermore, the theta value was estimated using the expected *a posteriori* method (EAP; Bock and Mislevy, 1982) according to their responses and item parameters. In the test stage, the Fisher information values for each remaining item were calculated. Then, at the temporary estimate of the new theta, the next item was chosen based on the maximum Fisher information criterion (Linden, 1998), which is one of the most frequently employed criteria for item selection in CAT. In the stop stage, CAT stopped when the standard error (SE) of theta achieved 0.447/0.386/0.316/0.224, which represented measurement reliabilities of 0.800/0.850/0.900/0.950, respectively. The final stage presented all the analysis results, including the final estimated theta, number of response items, and standard error of measurement (SEM). We used the R package “catR” (Magis and Barrada, 2017) for the analysis.

Furthermore, a real-data simulation of the CAT-ER was also conducted to check the quality of the item bank thoroughly. There is a difference between the real-data CAT-ER simulation and the simulated-data CAT-ER simulation. The former used the real 387 subjects’ responses collected before, whereas the latter employed the simulated subjects’ responses. Item parameters and the real 387 subjects’ responses were input by employing the real-data CAT-ER simulation. The CAT also stopped when the SE (θ) was 0.447/0.386/0.316/0.224.

In CAT, efficiency and reliability are important factors for performance quality. Marginal reliability is easy to use and dynamically monitors the reliability of CAT (Green et al., 1984). Generally, marginal reliability refers to a function of SEM, as illustrated in Eqs. 1, 2. The greater the marginal reliability was, the smaller the SEM was. Moreover, marginal reliability is equal to the average reliability for all participants (Wainer et al., 2000) and calculated as follows:

(1) $$M\,R=1-S\,E^{2}$$

(2) $$S\,E=\frac{\sum_{i=1}^{N}S\,E\,\left(\theta_{i}\right)}{N}$$

In Eq. 2, *N* is the total number of examinees, *i* is the specific subjects, and *SE*(θ_*i*_) is the standard error of subject *i* at the final estimated θ. In addition, marginal reliability increases with decreasing SE. In this article, several statistics were conducted to investigate the efficiency and reliability of CAT-ER, including the mean and standard deviation of the selected items, mean SE, marginal reliability, the mean and standard deviation of estimated theta, and Pearson’s correlations between the estimated theta with the stopping rule of None and the remnant stopping rules. The number of selected items with the marginal reliability for each subject was plotted under several stopping rules employing the R package “ggplot2” (Wickham, 2011).

Validity is another essential index of performance quality in CAT. Only when the evaluation result of the CAT-ER is similar to that of the calibration scale, CAT-ER can be considered effective. For that purpose, we used calibration-related validity to evaluate the similarity. The revised Life Orientation Test (LOT-R) was chosen as the calibration scale, as it has been widely used to assess optimism (Scheier and Carver, 1985; Scheier et al., 1995). Furthermore, we employed the Chinese version of LOT-R translated by Gu and Wang (2012) to implement the analysis of criterion-related validity of CAT-ER. LOT-R has 10 items, including four filling items, three positive items, and three negative items, all scored on a 5-point Likert-type scale. The total score was the general index of optimism tendency, while the positive and negative item scores were used to measure optimism and pessimism, respectively. The criterion-related validity of CAT-ER was assessed using SPSS version 23 (George and Mallery, 2016).

## Results

### Item Bank Construction of CAT-ER

#### Unidimensionality

Forty-five items were removed in the EFA since the factor loadings were <0.30 or not significant (*p* > 0.05). After excluding 45 items with low factor loadings or non-significance from the item bank, we ran the one-factor model CFA based on the remaining 109 items. Findings indicated an acceptable model fit in CAF (RMSEA = 0.088, CFI = 0.955, and TLI = 0.945). The findings suggested that the remaining 109 items (see Table 4) were sufficiently unidimensional. Consistent with these findings, the generated a high value (0.874) and the ECV also had a high value (65.4%) indicating the existence of a dominant general ER factor. For the remaining items, the GRM fit the data well, which is evidenced by absolute values of standardized residuals <1.96 for all items. The goodness of fit was not statistically significant (*M*_2_ = 7740.187, *df* = 496, *p* = 0.070), suggesting that the 109 items indicated a single construct.

#### IRT Model Selection

Of the three models, the GRM fitted the remaining items best, as it had the smallest −2LL, AIC, and BIC values, and a non-significant goodness of fit (*M*_2_ = 7740.187, *df* = 496, *p* = 0.070) (Table 3). Thus, the GRM was employed to analyze the final CAT-ER item bank.

**TABLE 3:** Model-fit indices.

Model	−2LL	AIC	BIC	*M*_2_
Graded Response Model	240,265.2	241,375.2	244,032.5	7,740.187
Generalized Partial Credit Model	243,782.4	244,892.5	247,549.5	7,936.263
Nominal Response Model	241,316.8	243,100.8	247,371.6	7,779.689

*−2LL, −2Log-Likelihood; AIC, Akaike’s information criterion; BIC, Bayesian information criterion; M_2_ statistic is one of the widely used indices to test the overall goodness of fit.*

#### Local Independence

A total of 29 pairs of items indicated local dependence based on their residual correlations >0.20 and Q3 values >0.36. One item has local dependence in three item pairs, and we removed this item in the three item pairs. Accordingly, 27 items were eliminated on account of local dependence (Table 4). The remaining items satisfied the local independence well.

**TABLE 4:** Reasons for stepwise exclusion of the items.

Excluded reasons	Excluded items
Unidimensionality	DERS-3, 5, 6, 11, 17, 18, 20, 21, 23, 32, 33, and 34 TMMS-2, 8, 15, 20, 22, 25, 26, 27, and 28 RESE-3 and 8 ERQ-2, 4, 6, 8, and 9 CERQ-3, 4, 6, and 16 NMR-2, 7, 9, 10, 11, 12, 13, 19, 22, 25, 26, 28, and 30
Local Independency	DERS-2, 7, 16, 22, 28, 29, 35, and 36 TMMS-5, 12, 13, 14, 18, and 19 RESE-9, 10, 11, and 12 ERQ-10 CERQ-9, 10, 11, 12, 13, and 14 NMR-1 and 3
**S*-χ^2^*	CERQ-19, 20, 21, 22, 23, 24, 25, 26, 27, 28, 29, 30, 31, 32, 33, 34, 35, and 36 NMR-5
DIF	None
Discrimination	None

#### Item Fit

Nineteen items had a poor fit to the GRM (*S*-χ^2^ < 0.01) (Table 4). After excluding these items, the remaining 63 items were reevaluated and showed a good fit (*S*-χ^2^ > 0.01).

#### Differential Item Functioning

None of items’ Δ*R*^2^ values were higher than 0.02 with corresponding *p* values higher than 0.05. Therefore, there was no DIF according to gender, age, or region for any items (Table 4).

#### Item Discrimination

A GRM was employed again to calibrate the remaining 63 items. The discrimination parameters of the remaining 63 items were all >0.50 with mean of 1.294 (*SD* = 0.371), indicating a high-quality item bank (Table 5).

**TABLE 5:** Item parameters for 63-item bank with GRM.

Item	a1	b1	b2	b3	b4	b5	b6
DERS-1	1.294	–3.020	–1.016	0.018	1.917		
DERS-4	0.943	–5.102	–3.054	–1.066	1.650		
DERS-8	1.243	–4.018	–1.667	–0.273	1.858		
DERS-9	0.971	–2.932	–0.738	1.179	4.294		
DERS-10	1.250	–3.649	–1.734	–0.108	1.979		
DERS-12	1.606	–1.987	–1.251	0.154	1.343		
DERS-13	0.771	–4.229	–2.116	–0.300	2.608		
DERS-14	0.977	–3.166	–1.064	1.024	3.151		
DERS-15	0.842	–3.609	–2.054	0.804	3.180		
DERS-19	0.642	–4.982	–1.985	0.980	3.364		
DERS-24	1.336	–2.791	–0.924	0.558	2.088		
DERS-25	0.898	–4.137	–1.353	0.892	3.389		
DERS-26	1.150	–3.242	–1.331	0.651	1.944		
DERS-27	1.582	–2.558	–1.015	0.412	1.329		
DERS-30	1.424	–2.092	–1.022	0.121	1.310		
DERS-31	1.406	–2.047	–1.325	–0.206	1.318		
TMMS-1	1.363	–3.201	–1.559	–0.075	1.527		
TMMS-3	1.019	–2.581	–0.683	0.675	2.394		
TMMS-4	1.782	–2.056	–0.838	0.084	1.175		
TMMS-6	0.881	–3.358	–1.512	0.324	1.609		
TMMS-7	0.786	–3.704	–1.791	0.340	1.809		
TMMS-9	0.989	–2.789	–1.339	0.380	1.470		
TMMS-10	0.823	–3.297	–1.433	0.686	2.365		
TMMS-11	1.086	–3.047	–0.938	0.583	2.642		
TMMS-16	1.166	–2.979	–0.695	0.823	3.121		
TMMS-17	0.926	–4.258	–1.766	0.142	2.249		
TMMS-21	1.212	–3.655	–1.699	–0.181	2.141		
TMMS-23	1.518	–2.949	–1.153	0.029	1.841		
TMMS-24	1.094	–2.865	–1.163	0.306	2.719		
TMMS-29	1.465	–2.654	–0.817	0.319	1.848		
TMMS-30	1.410	–2.615	–1.251	0.265	2.534		
RESE-1	1.181	–4.814	–2.626	–0.846	1.382		
RESE-2	1.668	–2.056	–1.298	–0.334	1.103		
RESE-4	1.907	–1.473	–0.672	0.210	0.925		
RESE-5	1.813	–1.348	–0.244	0.363	0.828		
RESE-6	1.967	–1.421	–0.195	0.438	1.053		
RESE-7	2.318	–1.262	–0.265	0.323	1.035		
ERQ-1	1.968	–2.802	–1.735	–0.820	0.042	0.861	2.071
ERQ-3	1.449	–3.979	–2.590	–1.367	–0.320	0.691	2.205
ERQ-5	1.469	–3.126	–1.813	–0.992	–0.085	0.883	2.043
ERQ-7	1.114	–4.805	–3.180	–2.030	–0.457	1.092	3.316
CERQ-1	1.212	–1.993	–0.539	1.155	3.135		
CERQ-2	1.401	–3.147	–1.584	0.079	2.011		
CERQ-5	1.137	–4.570	–2.636	–0.363	2.064		
CERQ-7	1.381	–2.222	–0.428	0.883	2.391		
CERQ-8	1.258	–4.348	–1.963	–0.078	2.603		
CERQ-15	2.220	–1.397	–0.442	0.274	0.992		
CERQ-17	1.206	–2.812	–1.414	–0.096	1.415		
CERQ-18	1.392	–2.750	–1.561	–0.343	1.257		
NMR-4	1.018	–4.053	–1.961	–0.221	2.641		
NMR-6	1.797	–2.233	–1.235	–0.261	1.509		
NMR-8	1.528	–1.886	–0.580	0.565	1.886		
NMR-14	0.968	–3.312	–0.775	0.948	3.457		
NMR-15	1.420	–3.008	–1.386	–0.085	2.226		
NMR-16	1.624	–1.922	–0.485	0.638	1.646		
NMR-17	1.645	–2.019	–1.004	0.219	1.417		
NMR-18	1.057	–3.383	–0.933	0.778	3.309		
NMR-20	0.972	–4.725	–1.871	0.267	3.473		
NMR-21	0.513	–6.190	–0.821	2.610	7.032		
NMR-23	1.031	–3.981	–1.651	0.052	2.567		
NMR-24	1.383	–2.128	–0.497	0.825	1.988		
NMR-27	1.606	–1.846	–0.328	0.847	1.958		
NMR-29	1.037	–4.010	–1.811	–0.014	2.874		

**a*, discrimination parameter; *b*, difficulty parameter.*

The above steps produced a 63-item set of CAT-ER (Table 5) that satisfied the unidimensionality and local independence hypotheses in IRT, fitted the GRM well, had high item discrimination parameters, good item fit, and no DIF. Furthermore, the real 387 subjects’ responses were used to validate the psychometric properties of the final item set of the CAT-ER. According to the one-factor CFA model of the remaining 63-item set, acceptable fit indices were obtained (RMSEA = 0.077, CFI = 0.902, and TLI = 0.913). In addition, the general ER factor of the bifactor model for all items accounted for 78.5% of the variance in the summed score and 63.5% of the common variance for all items. Thus, the common variance for all items mainly emerged from the general ER factor. These findings show that the final item set of the CAT-ER fully met the hypothesis of unidimensionality. In addition, the goodness of fit was not statistically significant (*M*_2_ = 5970.956, *df* = 693, *p* = 0.125), indicating that the final item set of the CAT-ER represented a single construct. Furthermore, none of the 63 items indicated local dependence (residual correlations: <0.20; Q3 values: <0.36) and the item fit evaluation suggest that all them fitted the GRM (*S*-χ^2^ > 0.01). None of the 63 items’ Δ*R*^2^ values exceeded 0.02, and the corresponding *p* values were all >0.05 in any of the DIF comparisons (gender, age, and region). Based on these analyses using the real 387 subjects’ responses, we confirmed that the items retained in the final CAT-ER item bank have acceptable item characteristics. This also increases the generalizability of study findings to replication studies using a smaller sample.

### Simulation of CAT-ER

#### Results Based on the Simulated Data of the CAT-ER

Table 6 shows the simulated data of the CAT-ER findings under different stopping rules. For these stopping rules, the mean number of selected items ranged from 5.135 to 26.079 or 8.2 to 41.4% of the full-item bank. These findings were satisfactory, particularly with the SE (theta) ≤ 0.447, the CAT-ER employed merely approximately five items while reaching the full-item efficiency. Moreover, with the SE (theta) ≤ 0.224, the CAT-ER saved more than half of the item bank usage. The mean SE of theta for several stopping rules ranged from 0.222 to 0.422. It reveals that corresponding measurement precision was achieved in each stopping rule. Furthermore, the marginal reliabilities under several stopping rules varied from 0.822 to 0.969, which were mostly acceptable for individuals. Table 6 also presents the Pearson’s correlations (*r*) between the CAT-ER theta estimates and full-item bank theta estimates under several stopping rules, which were all greater than 0.900.

**TABLE 6:** Simulated-data simulation statistics for CAT-ER under several stopping rules.

Stop rules	Number of selected items		% all	Mean SE (theta)	Marginal reliability	Estimated theta		*r*
	Mean	SD				Mean	SD	
None	63	0	100	0.176	0.969	0.017	0.982	1
SE (theta) ≤ 0.447	5.135	0.985	8.2	0.422	0.822	–0.037	0.924	0.915**
SE (theta) ≤ 0.386	6.698	1.462	10.6	0.374	0.860	–0.031	0.941	0.938**
SE (theta) ≤ 0.316	10.690	2.454	17.0	0.310	0.904	0.017	0.972	0.956**
SE (theta) ≤ 0.224	26.079	4.507	41.4	0.222	0.951	–0.039	0.951	0.975**

*None*, all item bank was used; % *all*, the percentage of the mean numbers of selected items in the full-item bank; *r*, the Pearson’s correlation; ** 0.01, level of significant correlation (two-tailed).

**FIGURE 1:**
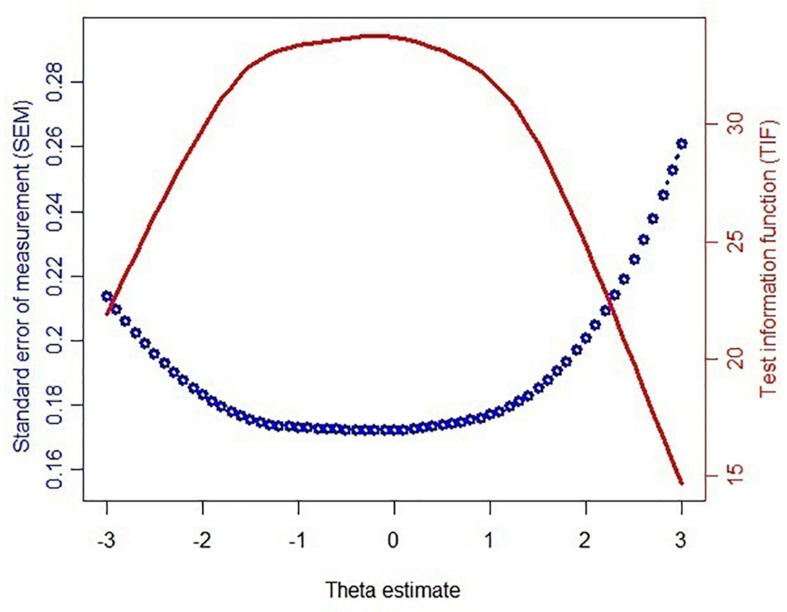
Test information function (TIF) and standard error of measurement (SEM) of the theta values estimated via 63 items of the bank.

Figure 1 reveals the findings of the test information function (TIF) and SEM across the theta under the simulated data of the CAT-ER. The greater the theta value, the greater the ER. More information denotes greater precision for measurement. A reliability coefficient of 0.85 or higher suggests that the measure has a good reliability (May et al., 2006). Naturally, the low-SEM criterion is a value of 0.39 or higher. These values are considered acceptable, because all the SEM values were under 0.39. The greater the TIF at each theta level, the smaller the SEM. According to the results, it is easy to see that the CAT-ER achieved adequate information and a reasonable standard error.

#### Results Based on Real Data of the CAT-ER

**FIGURE 2:**
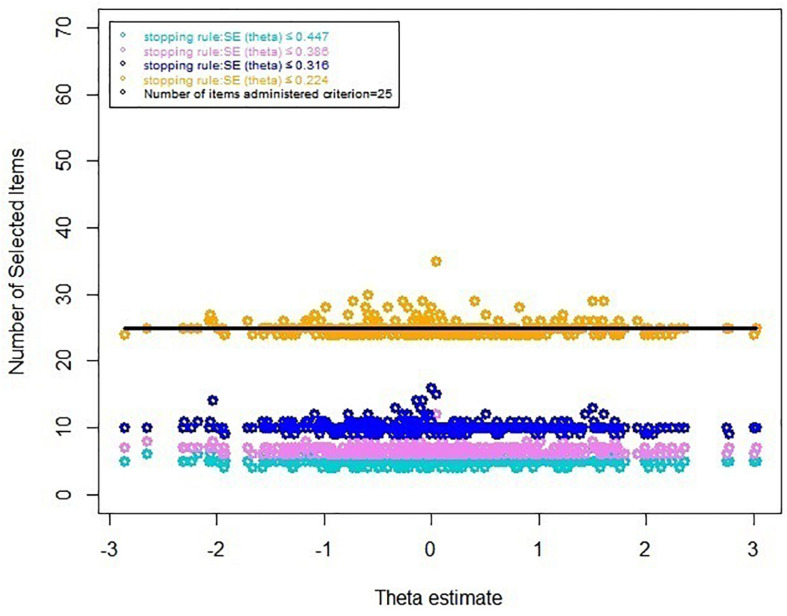
Number of selected items under several stopping rules.

Table 7 displays the results of the real data for the CAT-ER under several stopping rules. The results were consistent with the aforementioned results of simulated-data CAT-ER simulation. Moreover, the number of selected items in real-data simulation was even lower than that in the simulated-data simulation. Furthermore, we depicted an additional graph (Figure 2) that visually indicated the number of selected items across the theta under different stopping rules, illustrating that most items executed were below the horizontal line labeled as 25 items, that is, 39.6% of entire item bank. Overall, all these findings demonstrate the CAT-ER performed well in the real-data simulation.

**TABLE 7:** Real-data simulation statistics for CAT-ER under several stopping rules.

Stop rules	Number of selected items		% all	Mean SE (theta)	Marginal reliability	Estimated theta		*r*
	Mean	SD				Mean	SD	
None	63	0	100	0.173	0.970	−0.901	0.421	1
SE (theta) ≤ 0.447	4.953	0.475	7.9	0.423	0.821	−0.039	0.476	0.947**
SE (theta) ≤ 0.386	6.535	0.687	10.4	0.372	0.861	−0.042	0.491	0.901**
SE (theta) ≤ 0.316	10.137	0.848	16.1	0.309	0.904	−0.035	0.478	0.873**
SE (theta) ≤ 0.224	24.935	1.110	39.6	0.222	0.951	−0.037	0.421	0.891**

*None*, all item bank was used; % *all*, the percentage of the mean numbers of selected items in the full-item bank; *r*, the Pearson’s correlation; ** 0.01 level of significant correlation (two-tailed).

As shown in Table 8, the Pearson’s correlations between the CAT-ER and the LOT-R varied from 0.813 to 0.851 under several stopping rules, and there was statistically significant difference (*p* < 0.01). This means that regardless of the stopping rule used, the CAT-ER had acceptable criterion-related validity.

**TABLE 8:** Criterion-related validity of CAT-ER with external criteria scale under several stopping rules.

Stop rules	LOT-R
None	0.851**
SE (theta) ≤ 0.447	0.813**
SE (theta) ≤ 0.386	0.827**
SE (theta) ≤ 0.316	0.829**
SE (theta) ≤ 0.224	0.837**

*None*, all item bank was used; *LOT-R*, the revised Life Orientation Test; **0.01 level of significant correlation (two-tailed).

## Discussion

In this study, we developed a CAT-ER as a novel and effective assessment of ER. Psychometric evaluations were implemented in the initial ER item bank, and items were excluded until all the remaining items had favorable psychometric properties. Subsequently, the final CAT-ER item bank (based on two simulation studies) was evaluated for efficiency, reliability, and validity. The findings indicated that the final 63-item set satisfied the unidimensional and local independent assumptions, fitted the GRM well, had high item discrimination parameters, acceptable item fit, and no DIF. Furthermore, the proposed CAT-ER could greatly lessen the number of test items and thereby decrease the test burden on subjects, while also possessing desirable reliability and criterion-related validity. Specifically, there was no DIF based on gender, region, or age of subjects, which improves our confidence in advancing this CAT-ER version.

Researchers suggest that each measure mentioned above is formed based on the same underlying ER structure (Garnefski et al., 2001; Gross and John, 2003; Gratz and Roemer, 2004; Caprara et al., 2008). Furthermore, critical evidence from several studies on ER (Aldao et al., 2010; Gross, 2014; Naragon-Gainey et al., 2017; Megreya et al., 2018) indicates that each scale measures and evaluates the same underlying structure. Regarding empirical evidence, the one-factor CFA model and the dimensionality evaluation of IRT methods demonstrated that the final 63-item set of CAT-ER satisfied the hypothesis of unidimensionality. Consistently, a bifactor analysis suggested a dominant general ER factor that can be extracted in these ER items. The results further strengthen the usual practice of employing the total score of these measures as a general index of ER. The degree of correlations among test mean scores of these measures ranged from 0.66 to 0.83, which indicates moderate to high correlations (*p* < 0.01), suggesting that they measured the underlying ER structure. Overall, both theoretical and empirical findings suggest the same underlying structure (i.e., ER) for the self-report ER measures in this study. Furthermore, studies indicated that ER is a rather complex construct; the inconsistencies with respect to the precise number and composition of the subscales are main obstacles to carrying out multidimensional scoring structures for ER measures (Catanzaro and Mearns, 1990; Salovey et al., 1995; Garnefski et al., 2001; Gross and John, 2003; Gratz and Roemer, 2004; Caprara et al., 2008). Fliege et al. (2005) studied CAT on depression levels (D-CAT) and Walter et al. (2007) on anxiety levels (Anxiety-CAT), both single-dimensional CAT, even though depression and anxiety are both multidimensional. Based on the theoretical and empirical analyses, as well as common practices in previous studies, a unidimensional CAT-ER was developed to measure and assess the individual’s overall ER in the present study.

Compared with previous studies, this study has some unique advantages. First, methodologically, this study used CAT to assess ER by establishing an effective item bank based on IRT methods, whereas questionnaires were used in most previous studies to evaluate ER based on CTT methods. IRT accounts for parameter invariance, which can ensure that the result will be unaffected by other results regardless of whether the subject is from a representative sample. Accordingly, the CAT-ER can evaluate the individual’s ER precisely and effectively. Second, different test score systems can be compared in the CAT-ER. This approach ensures the difficulty of endorsing an item and the subjects’ ER level to be on the same scale. Third, although many screening measures are available, the agreement between them is less than optimal, and no measure can be deemed a gold standard (Garnefski et al., 2001; Gross and John, 2003; Garnefski and Kraaij, 2006; Nadia and Vivian, 2007; Caprara et al., 2008). It accordingly might be difficult for researchers and clinicians to select an optimal instrument while evaluating ER. However, based on this study, it is suggested that subjects’ ER can be estimated without the need to select a specific questionnaire.

This study demonstrated that the CAT-ER had acceptable efficiency, reliability, and validity. However, Figure 1 indicates that the CAT-ER offered little test information for those with a theta above 3 or below −3, implying that the estimation precision of the CAT-ER for these subjects is uncertain. Future studies could develop a CAT suitable for these subjects. In addition, in a real-life setting, subjects’ responses will be affected by many factors because the real-data simulation study may be different from a realistic environment (Smits et al., 2011). Based on the findings of a previous study (Kocalevent et al., 2009), the performance of CAT simulation was consistent with actual management findings, but it is uncertain that the similar outcome can be extended to an actual working CAT-ER program. Therefore, future studies could aim to design a CAT-ER for application in real-life settings.

Furthermore, several limitations should be considered in future studies. First, unidimensionality may not be fully satisfied in practice. For instance, many psychological and health-related tests are multidimensional measures. Multidimensional CAT or cognitive diagnostic CAT can be considered to treat multidimensional measures. Second, local dependency usually exists in some psychological or educational tests. The testlet model can be employed to address local dependency between items in future research. Third, the test developers still have much research to complete since the CAT procedure must be carefully conducted and maintained. Fourth, the maximum Fisher information item selection rule was selected in this study due to its popularity (Magis and Barrada, 2017). Future research should choose various item selection rules, for example, the global information item selection rule, to enhance measurement precision.

## Conclusion

The CAT-ER item bank had acceptable psychometric properties in the IRT and showed desirable performance in reducing the number of selected items without decreasing measurement accuracy. As a reliable and effective evaluation tool, the CAT-ER can evaluate crucial clinical or practical problems and help promote the development of effective ER interventions.

## Data Availability Statement

The original contributions presented in the study are included in the article/supplementary material, further inquiries can be directed to the first author.

## Ethics Statement

The studies involving human participants were reviewed and approved by the local Ethics Committee of School of Psychology, South China Normal University. The participants provided their written informed consent to participate in this study.

## Author Contributions

LX: data analysis and paper writing. RJ: data collection. FH and ZL: paper guidance. YZ: data collection. MZ: method guidance. All authors contributed to the article and approved the submitted version.

## Conflict of Interest

The authors declare that the research was conducted in the absence of any commercial or financial relationships that could be construed as a potential conflict of interest.
